# Use of F18 bioglass putty for induced membrane technique in segmental bone defect of the radius in rabbits

**DOI:** 10.1590/acb392424

**Published:** 2024-05-24

**Authors:** José Ivaldo Siqueira Silva, Sheila Canevese Rahal, Jennifer Gabriela Figueroa Coris, Bruna Martins da Silva, Felipe Cesar da Silva Brasileiro, Diana Nascimento, Zara Alves Lacerda, Jeana Pereira da Silva, Maria Jaqueline Mamprim, Marina Trevelin Souza

**Affiliations:** 1 Universidade Estadual Paulista – School of Veterinary Medicine and Animal Science – Department of Veterinary Surgery and Animal Reproduction – Botucatu (SP), Brazil.; 2 Universidade Estadual Paulista – School of Veterinary Medicine and Animal Science Department of Veterinary Clinics – Botucatu (SP), Brazil.; 3 Vetra – Produtos Cerâmicos de Alta Tecnologia – Ribeirão Preto (SP), Brazil.

**Keywords:** Histology, Bone Substitutes, Bone Development, Materials Testing

## Abstract

**Purpose:**

To evaluate the inductive capacity of F18 bioglass putty on the induced membrane technique in a segmental bone defect of the rabbit’s radius.

**Methods:**

Ten female Norfolk at 24 months of age were used. The animals were randomly separated based on postoperative time points: five rabbits at 21 and four at 42 days. A 1-cm segmental bone defect was created in both radii. The bone defects were filled with an F18 bioglass putty.

**Results:**

Immediate postoperative radiographic examination revealed the biomaterial occupying the segmental bone defect as a well-defined radiopaque structure with a density close to bone tissue. At 21 and 42 days after surgery, a reduction in radiopacity and volume of the biomaterial was observed, with particle dispersion in the bone defect region. Histologically, the induced membrane was verified in all animals, predominantly composed of fibrocollagenous tissue. In addition, chondroid and osteoid matrices undergoing regeneration, a densely vascularized tissue, and a foreign body type reaction composed of macrophages and multinucleated giant cells were seen.

**Conclusions:**

the F18 bioglass putty caused a foreign body-type inflammatory response with the development of an induced membrane without expansion capacity to perform the second stage of the Masquelet technique.

## Introduction

Masquelet’s technique has been applied in human patients for reconstruction of severe segmental bone defects, treatment of osteomyelitis, and infected or recalcitrant non-unions through a two-stage approach^1^^-^ 4. The first stage includes the induced membrane development after the debridement of tissues and the application of a polymethyl methacrylate (PMMA) cement spacer^1^^,^ 5. In the second stage of the procedure, the induced membrane forms a physical barrier, containing and preventing reabsorption of the autologous cancellous graft applied to the bone defect, but also stimulating its revascularization and corticalization since it is highly vascularized and secretes growth factors^6^^,^ 7.

Some disadvantages and complications of the technique have been reported, including: the requirement of two invasive surgical procedures that may pose risk in elderly patients; the need for reconstructive techniques in cases of insufficient soft tissue covering; the large amounts of autogenous bone graft that must be harvested in extensive segmental bone defect; requirement of further intervention due to inadequate evolution or re-fracture, among others^8^^,^ 9.

Several factors may influence the results, including the fracture immobilization method, bone graft harvesting technique, the size and location of the bone defect, and the care taken during the surgical procedure^10^. Thus, efforts have been made to improve technique outcome, such as the use of spacers impregnated or coated with antibiotics, the incorporation of biomaterials together with the cancellous graft to expand the volume, the use of growth factors for osteoconductivity improvement, and the use of other materials to substitute the PMMA cement spacer^9^^,^ 11 ^,^ 12.

Therefore, bioactive glasses constitute a subgroup of ceramic materials within bone tissue engineering, whose dissolution after implantation promotes a layer of hydroxyapatite on the surface of the glass, which interacts with the collagen fibrils and chemically bonds to host bone^13^. Several factors can interfere with the regenerative capacity of bioactive glass scaffolds, including the production method, composition, microstructure, and superficial area^14^.

A new bioactive glass composition, called F18 (that belongs to the SiO_2_–Na_2_O–K_2_O–CaO–MgO-P_2_O_5_ system), combined workability and high bioactivity, promoting the formation of a three-dimensional porous vitreous fibrous scaffold and other complex shapes^15^^-^ 17. F18 bioactive glass has proven to interact with both hard and soft tissues and has demonstrated interesting outcomes on bone healing and antimicrobial and biofilm control^15^^-^ 18.

Therefore, this study aimed to evaluate the capacity of F18 bioglass, in a putty form, to induce the formation of the membrane on the Masquelet technique. The hypothesis was that F18 bioglass putty could promote the formation of the induced membrane with histological characteristics similar to that already described for PMMA cement spacer. To the best of the authors’ knowledge, this biomaterial has not been used for this purpose.

## Methods

This study followed the guidelines for the care and use of laboratory animals and was approved by the Ethics Committee for the Use of Animals (CEUA - no. 187/2021) of our School.

### Animals and experimental design

A sample of ten female Norfolk rabbits, 24 months of age, and mean body weight of 4 kg (± 0.5 kg) were used. They were kept individually in separate cages, provided with commercial pelleted diet and water *ad libitum*. The animals were randomly separated based on postoperative time points: five rabbits at 21 days and five at 42 days.

### Anesthesia and surgical procedure

To perform the surgical procedure, each rabbit was premedicated via intramuscular with morphine (2 mg/kg) and midazolam (2 mg/kg IM). Following this, dissociative anesthesia was administered using a combination of ketamine (30 mg/kg IM) and xylazine (0.5 mg/kg IM). Anesthesia was maintained with isoflurane and oxygen under a facial mask. In addition, local blockade with bupivacaine (1 mg/kg) was performed.

**Figure 1:**
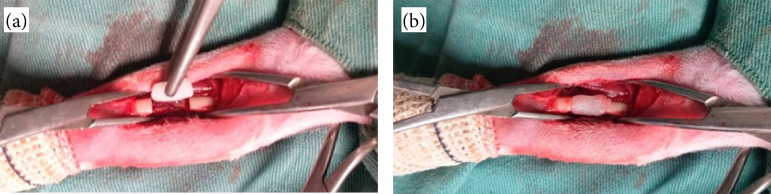
**(a)** F18 bioglass putty molded to be used in a 1-cm segmental radial defect in the rabbit. **(b)** Bone defect filled with F18 bioglass putty.

The rabbit was placed in dorsal recumbency, the hair on both forelimbs was clipped, and the skin was prepared for aseptic surgery. A craniomedial longitudinal incision of the skin and subcutaneous tissue was made in the middle-proximal third of the right radius. The extensor carpi radialis and common digital extensor muscles were retracted to expose the radius with periosteum. A 1-cm segmental bone defect was created with an oscillating saw. The bone defect was filled with an F18 bioglass putty provided by VETRA (Fig. 1). The putty was molded to fit the segmental bone defect at the moment of the surgery. The muscles and fascia were closed with a simple continuous pattern followed by a continuous intradermal skin closure using 3-0 polyglactin 910. The skin was closed using a simple interrupted suture 3-0 mononylon. Then, the same surgical protocol was applied to the left radius.

### Pre- and post-operative management and care

The animals were monitored after surgery until fully recovered from anesthesia. They received oxygen in a box and were warmed with an electric heating mat until the physiological parameters were normalized.

Immediately after the anesthetic induction and postoperatively, antibiotics (enrofloxacin; 10 mg/kg subcutaneously, every 24 hours, for five days), analgesics (tramadol hydrochloride; 10 mg/kg subcutaneously, every 12 hours, for five days), and anti-inflammatories (meloxicam; 1 mg/kg preoperatively and 0.5 mg/kg postoperatively for three days, subcutaneously) were administered.

The surgical incisions were evaluated and cleaned with normal saline and chlorhexidine digluconate every day. The skin sutures were removed on the 10th postoperative day. Besides, the animals were assessed after de surgery regarding comfort, forelimb load-bearing, food and water intake, defecation, and urination.

Radiographic images of the forelimbs were obtained in the craniocaudal and mediolateral projections, immediately after surgery and on postoperative days 21 and 42. A digital radiography system (SIUI, model SR 8100, maximum power 2 kW) was used at 80 cm focal-film distance with settings of 42 kV and 8 mAs. The position and radiopacity of the F18 bioglass in the segmental bone defect, and signs of bone growth (present or absent) were assessed.

### Histological and histochemical processing

Five rabbits were euthanized 21 days after surgery and four were euthanized 42 days after surgery to extract the induced membrane. One rabbit was excluded from the study because a fracture occurred in one operated forelimb. The procedure consisted of the intramuscular application of ketamine hydrochloride (50 mg/Kg) and xylazine hydrochloride (1 mg/Kg), followed by intravenous propofol (10 mg/kg) until loss of ocular movements and reflexes, supplemented with 19.1% potassium intravenously until cardiorespiratory arrest. The samples were fixed in 10% buffered formalin for 72 hours, followed by storage in 70% alcohol. The induced membranes were then dehydrated, clarified, and embedded in paraffin blocks. From paraffin blocks, 5-μm thick sections were cut, and stained with hematoxylin and eosin (H&E) and Masson trichrome.

H&E-stained slides were evaluated by semi-quantitative scoring in absent (-), mild (+), moderate (++), intense (+++), with emphasis on tissue composition (fibrocollagenous tissue, myxoid areas, cartilage), infiltrate inflammation (histiocytes, lymphocytes, eosinophils), vascularization, necrosis, and mineralization. Giant cells were determined to be either absent or present. Masson’s Trichrome stained slides were assessed for collagenous tissue presence in focal, multifocal, and diffuse patterns.

### Statistical analysis

The data obtained from HE samples were tested for normality using the Kolmogorov-Smirnov. The Wilcoxon test was used to compare independent samples. Non-parametric data (tissue composition, inflammation, vascularization, necrosis, mineralization, presence of giant cells) were compared between time points. The R programming language (version 3.5.3) was used.

## Results

### Clinical and radiographic assessments

Changes in food intake, defecation, or urination were not observed. Mild edema was found in surgical wounds, which resolved within a maximum of five days after the surgery. All wounds healed without complications. The animals showed load-bearing in both forelimbs.

**Figure 2:**
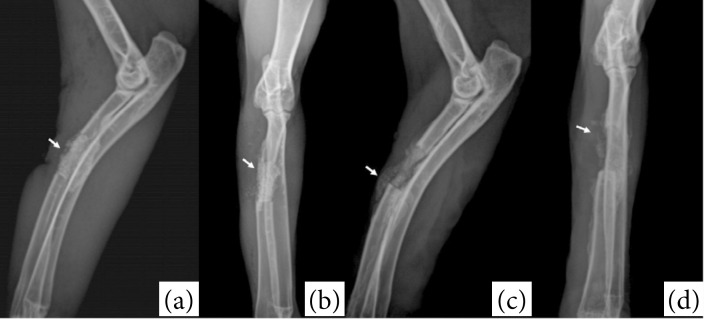
Mediolateral **(a, c)** and craniocaudal **(b, d)** radiographic views of the rabbit forelimbs. Observe well-defined radiopaque structure with a density close to bone tissue in the immediate postoperative **(a, b)**. Note a reduction in radiopacity and volume of the biomaterial at 42 days **(c, d)** after surgery.

Immediate postoperative radiographic examination revealed the F18 bioglass putty occupying the segmental bone defect as a well-defined radiopaque structure with a density close to bone tissue (Figs. 2a, 2b). At 21 and 42 days after surgery, a reduction in radiopacity and volume of the biomaterial was observed, with particle dispersion in the bone defect region (Figs. 2c, 2d). In addition, signs of bone formation were not seen at the fracture extremities. The size of the segmental defect did not change in the evaluation time points.

### Macroscopic and microscopic analyses

Macroscopically, the induced membrane had an aspect of delicate fibrous tissue with interconnected vitreous particles of biomaterial.

**Figure 3:**
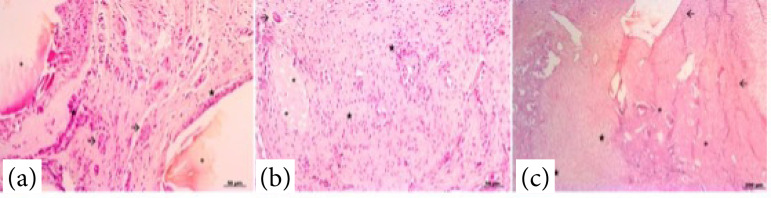
Photomicrographs of the induced membrane with F18 bioglass putty at 21 days after surgery. **(a)** Proliferation of intense, disorganized and highly vascularized fibrocollagenous tissue (arrows), and inflammatory infiltrate (star), encompassing vitreous particles (asterisks). HE, 20X. **(b)** Tissue composition predominantly of disorganized fibrocollagenous tissue, associated with intense mononuclear inflammatory infiltrate (star), focal areas of cartilage (asterisks), and presence of epithelioid cells and multinucleated giant cells (arrow). HE, 20X. **(c)** Presence of a pronounced chondroid matrix (star) and osteoid matrix (asterisks) in initial organization, fibrocollagenous tissue, and intense inflammatory infiltrate (arrows). HE, 5X.

**Figure 4:**
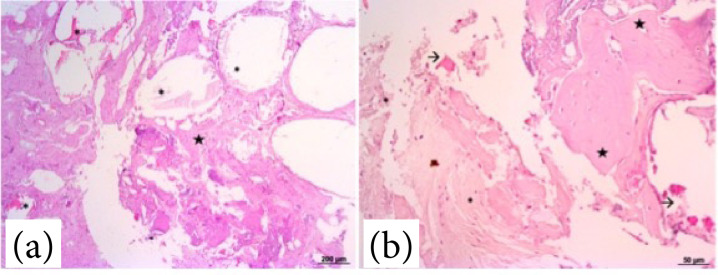
Photomicrographs of the induced membrane with F18 bioglass 42 days after surgery. **(a)** Proliferation of fibrocollagenous tissue with areas with glassy particles of F18 bioglass (asterisks). HE, 20X. **(b)** Proliferation of disorganized fibrocollagenous tissue and mononuclear inflammatory infiltrate (star) encompassing areas containing F18 bioglass (asterisks). HE, 40X. **(c)**. Intense and well-organized osteoid matrix (stars), and presence of loose fibrocollagenous tissue (asterisks). HE, 20X.

The F18 bioglass was still present in certain regions of the HE-stained samples 21 days after surgery. . There was tissue proliferation, inflammatory infiltration, and vascularization (veins and arteries), which encompassed vitreous particles (Fig. 3a). The layers in the induced membrane were not identified. The predominant tissue composition was fibrocollagenous tissue (70% intense) with focal areas of cartilage and presence of reactive fibroblasts, and moderate to intense vascularization (Fig. 3b). An intense mixed inflammatory infiltrate, composed mainly of histiocytes, lymphocytes and eosinophils, and a moderate amount of multinucleated giant cells were observed. In addition, areas of the chondroid matrix and osteoid matrix were detected (Fig. 3c). At 42 days after surgery, the samples had few areas containing the F18 bioglass particles (Fig. 4a). The predominant tissue composition was fibrocollagenous tissue (intense) with extensive areas of chondroid matrix and osteoid matrix across all sample regions, and moderate to discrete vascularization (Fig. 4b). A discrete inflammatory infiltrate characterized mainly of histiocytes, and rare multinucleated giant cells were found.

No statistically significant differences occurred between time points regarding fibrocollagenous tissue (p = 0.62) and myxoid areas (p = 0.80), and cartilage (p = 1). No statistically significant difference occurred between time points regarding inflammatory infiltrate as follows: histiocytes (p = 0.05), lymphocytes (p = 0.6578), and eosinophils (p = 0.7791). Vascularization intensity showed a statistically significant difference between time points (p = 0.005264), with greater vascularization intensity on day 21 after surgery. Multinucleated giant cells did not present a statistical difference between time points (p = 0.1294).

**Figure 5:**
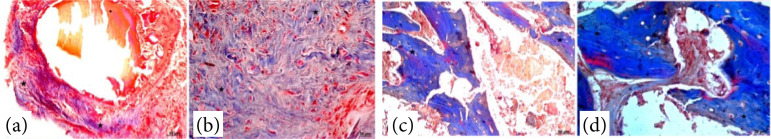
Photomicrographs of the induced membrane with F18 bioglass putty stained with Masson trichrome at 21 days **(a,b)** and 42 days after surgery **(c,d)**. **(a)** Focal areas of collagen fibers stained blue interspersed with fibrous tissue (star), and presence of vitreous particles (asterisks), 20X. **(b)** Diffuse presence of disorganized collagenous tissue (star), 40X. **(c)** Areas of chondroid matrix marked diffusely (star), 20X. **(d)** Proliferation of chondroid matrix and osteoid matrix both well-organized (star), 40X.

The histochemical analysis (Masson trichrome) showed connective tissue in different organization processes at both time points. At 21 days after surgery, there was a diffuse presence of disorganized collagenous tissue, with multifocal and diffuse areas in all analyzed samples (Figs. 5a, 5b). At 42 days after surgery, organized and dense connective tissue with diffuse blue markings was noted, mainly evidencing the intense presence of osteoid and chondroid matrices (Figs. 5c, 5d).

## Discussion

The current research found that the F18 bioglass putty promoted an induced membrane, but some histological characteristics differed from those described with a PMMA cement spacer.

Based on the clinical and histological evaluations, the F18 bioglass putty was safe in bone defects, considering that signs of rejection or infection were not observed. Although bone immobilization was not done with implants, bone defect size was not changed radiographically at time point evaluations because of intact ulna. The same phenomenon was verified in another study using a similar methodology but employing a PMMA cement spacer^19^. A fact to be considered is that the F18 bioactive bioglass putty does not have mechanical resistance^20^, which may be a problem in clinical cases. One of the functions of the PMMA spacer is to prevent soft tissue collapse into bone defect after debridement^7^^,^ 9 ^,^ 21 since it constitutes a solid element after highly exothermic polymerization^22^.

Radiographic scans at 21 and 42 days after surgery did not show signs of bone formation at the fracture extremities. A different study with radius segmental defect in young rabbits (3 months of age) under the influence of low-level laser therapy already verified bone growth from the cut ends just three weeks after the postoperative treatment. The impact of the animal age must be considered in this study since the rabbits were 24 months of age.. The radiographic changes in F18 bioglass putty suggested its biodegradation. Scanning electron microscope evaluation of the F18 bioactive fibrous glassy scaffold after phosphate-buffered saline incubation suggested degradation over time accompanied by ruptures in the fiber structures^16^.

Macroscopically, the vitreous particles of F18 bioactive bioglass putty were interconnected with the induced membrane in both time points, which makes it difficult to perform the second stage of the Masquelet technique. During the second stage, the induced membrane is cut open to removed the spacer^1^^,^ 5 ^,^ 7 and fill the cavity with either cancellous autograft bone graft or bone substitutes^5^^,^ 7 ^,^ 23. It is important to note that bioglasses bind to the bone by forming a hydroxyapatite layer on its surface when dissolved in the body fluid, thus stimulating cell proliferation and differentiation^17^. Therefore, further studies are required to evaluate if, in the long term, only F18 bioactive bioglass putty can repair the bone defect.

Histologically the induced membrane with the F18 bioactive bioglass putty did not show different layers. In turn, the induced membrane using PMMA spacer may have up to three layers, the inner portion composed of epithelial-like cells, the intermediate layer formed mainly of collagen fibers, and the outer portion presenting fibroblasts, myofibroblasts and collagen bundles^9^^,^ 19 ^,^ 24 ^,^ 25. In the current study, the induced membranes were histologically (HE stain) similar in both time points, with a predominance of fibrocollagenous tissue, and areas of chondroid and osteoid matrices. However, a better organization of the connective tissue was verified in histochemistry on day 42 compared to day 21 after surgery.

The inflammatory infiltrate in the membrane induced with F18 bioactive glass putty contained histiocytes (tissue macrophages), lymphocytes, eosinophils, and also multinucleated giant cells. The findings are consistent with a foreign body response, which is one of the main characteristics of the induced membrane with PMMA spacer^1^^-^ 3 ^,^ 9. The accumulation of monocytes or macrophages may be related to the secretion of inflammatory cytokines and chemokines, which recruit and attract inflammatory cells around PMMA^26^.

Statistically, the current study showed a greater intensity of vascularization in the induced membrane at 21 days after surgery. The maximal neovascularization of induced membrane in rats with PMMA spacer also occurred between 2 and 4 weeks and subsided by 6 weeks^27^. Histological and immunohistochemical evaluations have shown that the induced membrane with PMMA spacer is richly vascularized in all its layers and has several growth factors, such as VEGF, TGF-β1, and inductive factors such as BMP-2^1^^,^ 9 ^,^ 21 ^,^ 24. These factors contribute to graft incorporation in the second stage. The bioactive fibrous glassy scaffold (F18) applied to rat tibia defect (3 mm) showed progressive degradation of the material and its replacement by granulation tissue and woven bone. Also, F18 induced the expression of growth factors such as RUNX-2, RANK-L, and COL-1 confirming its bioactive and osteopromotive properties^16^.

Some limitations observed herein need to be addressed and clarified in future studies. The biomaterial evaluation with another type of presentation to promote the induced membrane and maintain the framework for the second stage of the Masquelet technique is one of them. The association of biomaterials to overcome this mechanical limitation found in bioglass is another one.

## Conclusion

The uses of F18 bioglass putty caused a foreign body-type inflammatory response with the development of an induced membrane without expansion capacity to perform the second stage of the Masquelet technique.

## Data Availability

All dataset were generated or analyzed in the current study.
